# Feasibility and Acceptability of Wearable Cameras to Assess Self-care in People With Heart Failure: Pilot Study

**DOI:** 10.2196/40536

**Published:** 2023-02-17

**Authors:** Sanna Askman, Marie Löf, Ralph Maddison, Rebecca Nourse

**Affiliations:** 1 Department of Health, Medicine and Caring Sciences Linköping University Malmö Sweden; 2 Department of Health, Medicine and Caring Sciences Linköping University Linköping Sweden; 3 Institute for Physical Activity and Nutrition Deakin University Geelong Australia

**Keywords:** heart failure, HF, life logging, self-management, cardiovascular disease, wearable devices, self management, self-care

## Abstract

**Background:**

Heart failure (HF) is a common chronic condition that affects over 26 million people worldwide. It is a progressive and debilitating disease with a broad symptom profile, intermittently marked by periods of acute decompensation. People with HF generally do not self-manage their condition well (eg, monitoring symptoms, taking medications regularly, physical activity, etc). A better understanding of self-care activities and what factors may indicate deterioration is warranted.

**Objective:**

The aim of this study was to determine the feasibility and acceptability of using wearable cameras to assess self-care activities in people with HF. The study objectives were to (1) explore whether changes in self-care activities could be identified prior to hospitalization and (2) determine the acceptability of wearable cameras to people with HF.

**Methods:**

A total of 30 people recently diagnosed with HF wore a camera for a maximum of 30 days; the camera took a photo every 30 seconds in the forward-facing direction. At the end of the study, all 30 participants were presented with 8 statements of acceptability, scored on a 5-point Likert scale. To determine whether camera images could identify changes in self-care activities and lifestyle risk factors before hospitalization, we analyzed images from participants (n=8) who were hospitalized during the 30-day study period. Images from the period immediately prior to hospitalization and a comparison were selected for each participant. Images were manually coded according to 9 different event categories relating to self-care and lifestyle risk factors, and events were compared between the 2 periods.

**Results:**

The participants reported high acceptability for wearing the cameras, as most strongly agreed or agreed that they were comfortable to wear (28/30, 93%) and easy to use (30/30, 100%). The results of the camera image analysis showed that participants undertook fewer activities of daily living (*P*=.008) and were more sedentary (*P*=.02) prior to being hospitalized, compared to a period nonadjacent to hospitalization.

**Conclusions:**

Adults with HF were accepting of using a wearable camera for periods within a 30-day time frame. Wearable cameras were a feasible approach for providing data on selected self-care activities and lifestyle risk factors for HF and offer the potential to be a valuable tool for improving our understanding of self-care.

## Introduction

### Heart Failure—A Public Health Problem

Heart failure (HF) is a progressive and debilitating condition characterized by symptoms such as tachycardia, dyspnea, and fatigue caused by insufficient pumping of blood from the heart [1], which leads to hemodynamic and neurohormonal alterations [2]. Worldwide, HF affects more than 26 million people [3], and morbidity, mortality, and hospitalization rates remain high [4]. Approximately 1%-3% of total health care costs in Western Europe, North America, and Latin America are attributed to HF, with most of these costs due to hospitalizations [3]. However, it is estimated that half to two-thirds of HF hospitalizations are preventable [5]. One component of HF management that is integral to reducing hospital admissions, as well as decreasing the risk of mortality and maintaining quality of life in people with HF, is effective patient self-care [6,7].

### Self-care—A Key Feature in HF Treatment

Self-care refers to the day-to-day management of chronic conditions by individuals to maintain wellness (physical and mental) and control symptoms and illness progression. It refers to a range of activities such as adhering to prescribed medications, undertaking daily care activities (eg, blood glucose monitoring, self-weighing, rehabilitation exercises, and toileting activities), managing body weight (eg, reducing energy intake and increasing physical activity), and managing a healthy dietary intake (eg, limiting salt consumption) [8]. Despite the importance of self-care, several studies have reported ineffective practices such as poor symptom recognition and delayed symptom reporting [9,10]. Additionally, nonadherence to medications and positive lifestyle habits change is common, with 60%-80% of people with HF failing to follow recommendations [11,12].

The EuroHeart Failure Survey [13] showed that recall and adherence to self-care advice in people living with HF were poor but suggested that a detailed picture of their self-care could help understand where the problems lie [13]. Health care professionals, such as HF nurses, usually base their understanding of self-care and subsequent advice on client interviews, sometimes supplemented by nurse home visits. However, this does not fully capture the daily activities and challenges of people living with HF and may be subject to recall bias [14]. Improving health care professionals' understanding of an individual's self-care activities could optimize HF management and reduce future hospital admissions [8].

### Wearable Cameras—A Technology to Aid Self-care Research and Interventions

Increasingly, technologies have been harnessed to better understand and support self-care activities in people with a range of health conditions. One such technology is visual “life-logging,” which refers to the use of wearable cameras to digitally capture everyday life activities through first-person point-of-view images [15]. Wearable cameras offer benefits for assessing self-care as they address some limitations of existing techniques [14]. For example, the fact that a researcher is not present during data collection is beneficial as it increases the likelihood of obtaining an accurate description of the participant’s behavior, and recall bias associated with self-report is reduced [16,17]. Recent technological developments involving prolonged battery time and miniaturization of the cameras have allowed this technology to be used for research on sedentary behavior and physical activity [18-20], as well as dietary habits [21-23]. A scoping review of evidence on the use of wearable cameras in self-care research found that they are predominantly used to capture health-related behaviors (eg, dietary intake and exercise) but not specific activities important in chronic disease self-care (eg, taking medication and self-weighing) [24]. Indicating potential for use in self-care interventions, wearable cameras have been used as a memory aid for people with Alzheimer disease and cognitive impairment, and in rehabilitation training for people with acquired brain injury [25-27].

In summary, wearable cameras may offer a potential tool to assist HF management. An enhanced picture of an individual’s self-care activities could augment existing health care services and provide new avenues for interventions. However, as no studies have used wearable cameras in people with HF for this purpose, the feasibility of detecting specific self-care activities using images from wearable cameras and acceptability in this population need to be determined.

### Study Aim

The aim of this study was to determine the feasibility and acceptability of using wearable cameras to assess self-care activities in people with HF. The study objectives were to (1) explore whether changes in self-care activities and lifestyle risk factors could be identified prior to hospitalization and (2) determine the acceptability of wearable cameras to people with HF.

## Methods

### Study Design

**Figure 1:**
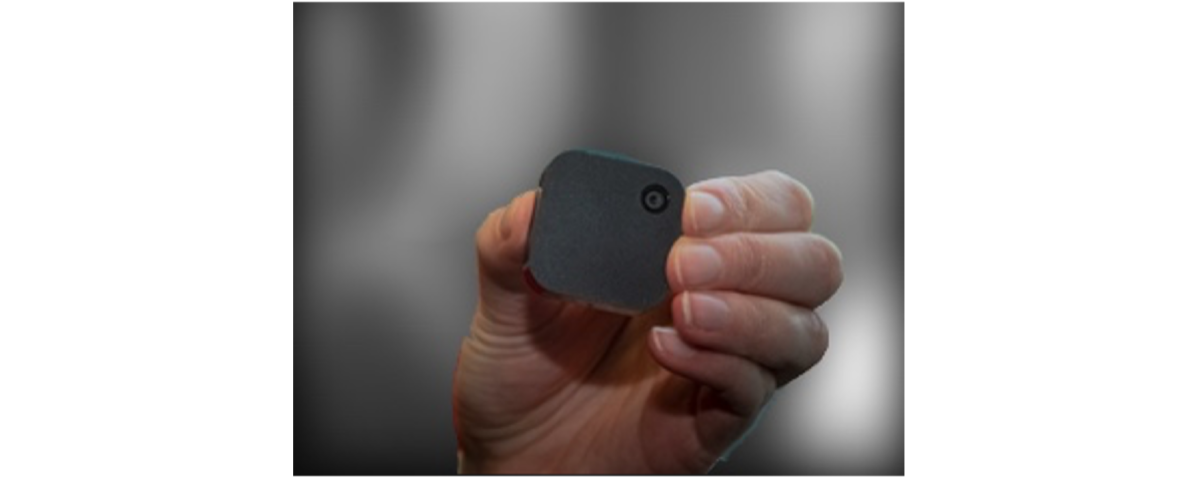
The Narrative Clip wearable camera.

This study was part of a larger prospective observational pilot study that aimed to determine the feasibility and acceptability of using wearable cameras and point-of-care testing to assess self-care activities and identify periods of decompensation in people with HF. The study involved 30 people diagnosed with HF who wore a wearable camera for a maximum of 30 days and underwent point-of-care testing and self-assessed symptom scoring twice a week during the study period (Figure 1). In this study, we report on the feasibility of using wearable camera images to identify changes in self-care activities and lifestyle risk factors between the period immediately prior to hospitalization (T1) and a comparison period (T2) for participants who were readmitted to hospital during the 30-day study period (n=8). The acceptability of wearable cameras to all participants (N=30) is also reported. The feasibility of point-of-care testing is reported elsewhere [28].

### Study Population, Recruitment, and Setting

Participants were recruited from a single-center HF outpatient clinic at a tertiary hospital in Melbourne, Australia. Inclusion criteria were newly diagnosed HF (New York Heart Association, functional class II or III) outpatients, older than 18 years, able to read and understand English, and treated with medication. Participants were approached by a cardiologist or a trained researcher during outpatient clinics and asked to participate. Those who agreed were contacted by a researcher from Deakin University, who explained the study and undertook screening to determine eligibility. Eligible participants were scheduled for a baseline assessment, at which time a written consent was obtained.

### Study Procedures

Participants were asked to wear the Narrative Clip camera (Figure 1) on their shirts during waking hours over a maximum period of 30 days. The Narrative Clip camera is a small wearable life-logging camera that automatically takes an image in the forward-facing direction every 30 seconds. At the baseline assessment, participants were informed on how to attach the camera to their shirt and charge it overnight. Participants were asked to carry out their regular activities during the study period; no advice to modify lifestyle behavior was given by the research team. Medical history, demographic data, and physical measurements were obtained at the baseline assessment. The research team visited participants twice weekly during the study period to download images, at which time participants were given the opportunity to delete images. Physical parameters were also monitored at the twice-weekly visits; the results were reported elsewhere [28]. At the end of the study period, a short interview was conducted with each participant to ask about their experience using a wearable camera.

### Data Collection and Analysis

#### Self-care Activities—Event Detection

As this study aimed to determine the feasibility of using wearable cameras, the analysis was exploratory and descriptive in nature. As highlighted above, this analysis focused on only those (n=8) participants who were readmitted to hospital during the 30-day study period. Images from at least 2 days immediately prior to hospitalization (T1) were a requirement for inclusion in this analysis. The comparison period (T2) for each participant was at least 2 days the same days of the week as T1 and selected depending on when in the study the participant was hospitalized (2 weeks before hospitalization or 2 weeks after discharge).

Images from the selected days for each participant were sequentially reviewed and coded by 1 author (SA) who had received training from 2 authors (RM and RN) experienced with camera image review. Many images were similar; therefore, images were scanned until a unique event (an image capturing an event of interest) was observed.

The research team developed categories for the events of interest after reviewing published literature on self-care and lifestyle risk factors [8]. The following 9 categories were identified: (1) sedentary behaviors—sit and eat, sit and screen (eg, television and computer), and sit and other tasks (eg, doing crosswords and reading); (2) physical activity (eg, cleaning, gardening, and walking); (3) activities of daily living (ADL; eg, cooking or washing); (4) presence of packaged foods; (5) weight monitoring; (6) other activities (eg, going to church); (7) shopping; (8) driving; and (9) taking medication. An event was determined by a sequence of 5 or more images (equating to 2 minutes) except for events in categories that were not time sensitive (eg, the presence of packaged foods, weight monitoring, and taking medication); these only required 1 image.

**Figure 2:**
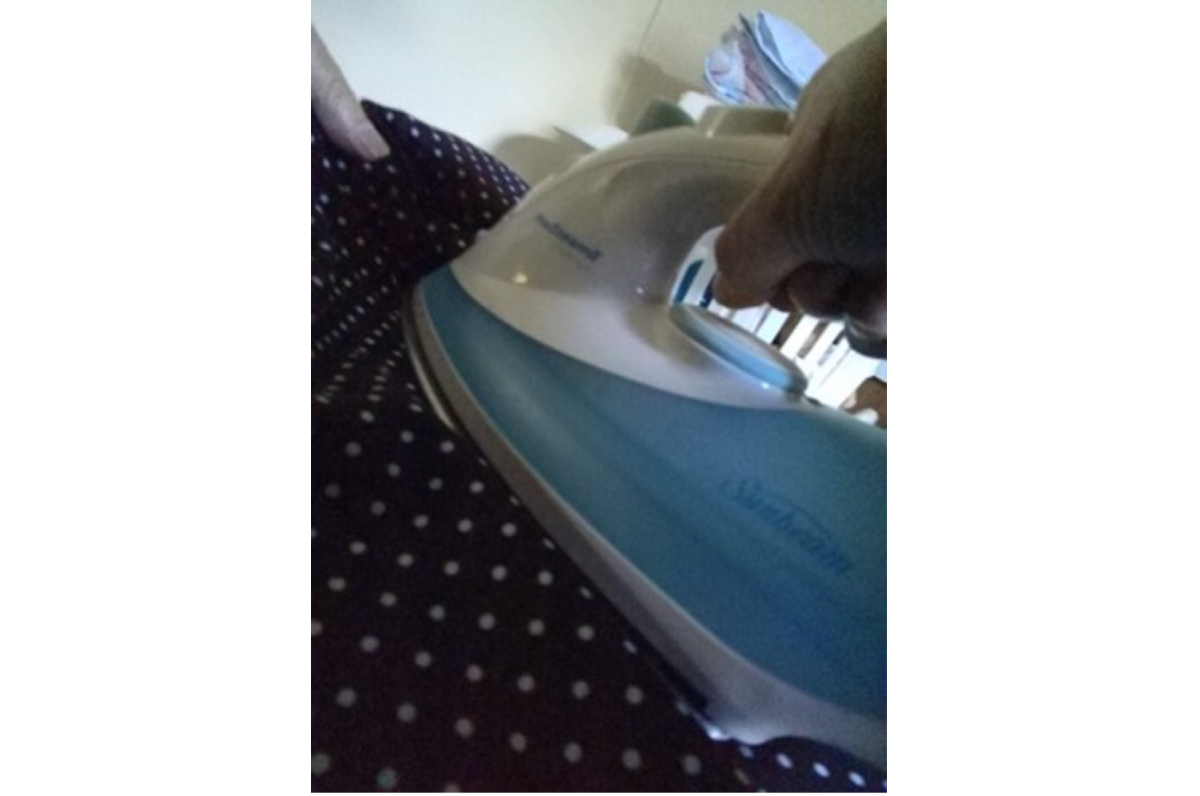
Example wearable camera image (participant using an iron).

All unique events were coded into these categories using an Excel worksheet (Microsoft Corporation), and the image reference was noted for ease of future reference. For example, the image in Figure 2, showing a participant using an iron, would be coded as ADL. Events were coded into a single category except for images displaying the presence of packaged foods, which could occur alongside an activity category (eg, ADL and cooking). The Excel worksheet contained information about the number of unique events in each category per day for each participant but did not capture time spent on the activities.

#### Acceptability

To assess acceptability, participants were presented with 8 statements to determine their perceptions of wearing the camera, including personal feelings, its utility, and technical issues. A 0-4 Likert-type scale (0=strongly disagree, 1=disagree, 2=neutral, 3=agree, and 4=strongly agree) was used to measure to what extent the participants agreed with the statements.

### Statistical Analysis

All data were exported from Excel to SPSS version 27 (IBM Corporation). The number of coded events from the 2 periods (T1 and T2) were tested for normal distribution using the Kolmogorov-Smirnov test. Thereafter, two-tailed paired sample *t* tests were used for comparison; significance level was at .05.

### Ethical Considerations

Ethical approval was obtained from the Human Research Ethics Committee at Deakin University (HREC/16/MH/55) and the Western Health Human Research Ethics Committee (2016.071). The participants received information about the study through a patient information sheet and verbal explanation from the research team. They were informed that they could leave the study at any time (but data collected to that point would be retained for analysis). All participants had an opportunity to ask questions about the study before they gave written informed consent. During the study, participants were given the opportunity to delete images downloaded from their wearable camera. On completing the study, participants received a AU $40 (US $28) voucher.

## Results

### Participant Characteristics

Of the 30 participants involved in the study, 10 (33%) were readmitted to hospital during the 30-day study period (Table 1).

Of the 10 participants readmitted to hospital with an exacerbation of HF, 2 (20%) did not wear the camera in the days prior to hospitalization and therefore did not meet the inclusion criteria for this analysis; thus, data from 8 participants were included. From these 8 participants, a total of 44 days of camera data were selected, and on average, 1000 images per day were available for each participant (total=44,000 images). For 6 participants, 6 days of camera data were analyzed, consisting of 3 days immediately prior to admission (T1) and 3 days from 2 weeks before hospitalization (T2). Moreover, 2 participants were hospitalized 2 days after the study commenced, and therefore only 4 days of camera data were analyzed for these participants—the 2 days immediately prior to admission (T1) and 2 days from 2 weeks after discharge (T2). All analyses included images from at least one weekday and a weekend day.

**Table 1:** Characteristics of the study participants (N=30).

Characteristics			All participants (N=30)	Not hospitalized (n=20, 66%)		Hospitalized (n=10, 33%)
Age (years), median (IQR)			77 (67-83)	73.0 (66-82)		80.5 (73-87)
**Sex, n (%)**						
	Male	18 (60)		12 (60)	6 (60)	
	Female	12 (40)		8 (40)	4 (40)	
**NYHA^a^ class, n (%)**						
	II	10 (33)		7 (35)	3 (30)	
	III	20 (66)		13 (65)	7 (70)	
**Years since HF^b^ diagnosis, n (%)**						
	≤5	18 (60)		12 (60)	5 (50)	
	5-10	3 (10)		2 (10)	1 (10)	
	10-20	7 (23)		5 (30)	2 (20)	
	>20	2 (7)		0 (0)	2 (20)	

^a^NYHA: New York Heart Association.

^b^HF: heart failure.

### Identified Events

Across all images selected for this analysis, 904 unique events were identified. Of these 904 events, 432 (48%) were sedentary behavior events, such as sitting or lying down, and 275 (30%) were activities of daily living, such as ironing or cooking. Physical activity events (eg, gardening) that occurred in the home environment (n=61, 7%) were more common than those outside the home (n=10, 1%; Table 2); however, 3 participants generated 75% (46/61) of home-based physical activity events. Of the 10 physical activity events that occurred outside the home, 1 (10%) was categorized as strenuous as the participant was exercising at a gym, and 9 (90%) were categorized as discrete walking events. No images in this analysis revealed participants monitoring their weight.

**Table 2:** Total events identified in each category (N=904).

Event category	Events identified, n (%)	Immediately prior to hospitalization (T1) (n=364, 40%), n (%)	Comparison period (T2) (n=540, 60%), n (%)
Sedentary behavior	432 (48)	189 (52)	243 (45)
ADL^a^ (at home)	275 (30)	89 (24)	186 (34)
Physical activity (at home)	61 (7)	27 (7)	34 (6)
Driving	45 (5)	19 (5)	26 (5)
Medication	39 (4)	14 (4)	25 (5)
Shopping	21 (2)	11 (3)	10 (2)
Packaged foods	21 (2)	9 (2)	12 (2)
Other physical activities (outside home)	10 (1)	6 (2)	4 (1)
Weight monitoring	0 (0)	0 (0)	0 (0)

^a^ADL: activities of daily living.

### Differences in Events Immediately Prior to Hospitalization (T1) and the Comparison Period (T2)

From the 904 events identified, 364 (40%) were in T1 and 540 (60%) in T2. In T1, participants had fewer unique events of sedentary behavior and ADLs compared to T2, indicating that they may have been sedentary for longer continuous periods of time prior to hospitalization. The number of medication events was lower in T1 (n=14) compared to T2 (n=25), and events relating to physical activities outside the home, shopping, and the presence of packaged foods were about the same across the 2 periods. Of the event categories, only sedentary behavior events (mean 23.6 vs 30.4 events; *t*=–3.11*, P*=.08) and ADL events (mean 11.1 vs 23.6 events; *t*=–3.70, *P*=.01) were significantly different between T1 and T2 (Table 3).

**Table 3:** Unique events in each category and comparison by time period.

Category	Immediately prior to hospitalization (T1; n=364), mean (SD)	Comparison period (T2; n=540), mean (SD)	*t* test	*P* value
Sedentary behavior	23.63 (5.71)	30.38 (10.39)	–3.11	.02
ADL^a^ (at home)	11.13 (8.64)	23.25 (17.12)	–3.70	.01
Physical activity (at home)	3.38 (4.00)	4.25 (3.65)	–2.198	.06
Driving	2.38 (2.56)	3.25 (3.15)	–1.31	.23
Medication	1.75 (1.67)	3.13 (2.95)	–2.31	.054
Shopping	1.38 (1.41)	1.25 (1.04)	0.28	.78
Packaged foods	1.13 (1.36)	1.5 (1.51)	–0.70	.50
Other physical activities (outside home)	0.75 (1.16)	0.5 (0.76)	0.80	.45
Weight monitoring	0.00	0.00	—^b^	—

^a^ADL: activities of daily living

^b^Not available.

### Acceptability

All participants (N=30) were presented with 8 statements of acceptability, scored on a 5-point Likert scale. Overall, participants reported high acceptability for wearing the cameras (Table 4).

**Table 4:** Statements to assess acceptability of the wearable cameras (N=30) obtained by means of interviews.

Statement		Value, n (%)
**The wearable camera is easy to use**		
	Strongly agree	29 (96.7)
	Agree	1 (3.3)
	Neutral	0 (0)
	Disagree	0 (0)
	Strongly disagree	0 (0)
**I feel comfortable when using the camera**		
	Strongly agree	25 (83.3)
	Agree	3 (10.0)
	Neutral	2 (6.7)
	Disagree	0 (0)
	Strongly disagree	0 (0)
**I feel comfortable when other people ask me about the camera**		
	Strongly agree	26 (86.7)
	Agree	2 (6.7)
	Neutral	2 (6.7)
	Disagree	0 (0)
	Strongly disagree	0 (0)
**I remember to wear my camera every day**		
	Strongly agree	16 (53.3)
	Agree	12 (40.0)
	Neutral	2 (6.7)
	Disagree	0 (0)
	Strongly disagree	0 (0)
**I initiate good behavior while wearing the camera**		
	Strongly agree	17 (56.7)
	Agree	12 (40.0)
	Neutral	1 (3.3)
	Disagree	0 (0)
	Strongly disagree	0 (0)
**I like to use new technology**		
	Strongly agree	6 (20.0)
	Agree	15 (50.0)
	Neutral	8 (26.7)
	Disagree	1 (3.3)
	Strongly disagree	0 (0)
**I have privacy when using the camera**		
	Strongly agree	6 (20.0)
	Agree	24 (80.0)
	Neutral	0 (0)
	Disagree	0 (0)
	Strongly disagree	0 (0)
**The camera can help people like me in the future**		
	Strongly agree	10 (33.3)
	Agree	18 (60.0)
	Neutral	2 (6.7)
	Disagree	0 (0)
	Strongly disagree	0 (0)

## Discussion

### Principal Results

This study sought to determine the feasibility and acceptability of wearable cameras to assess self-care activities and lifestyle risk factors in people with HF. Overall, the technology was considered feasible for better understanding self-care; however, technical issues warrant future attention. The technology was also acceptable to participants.

### Feasibility of Using Wearable Cameras

We demonstrated the feasibility of using wearable cameras to collect data on self-care activities and lifestyle risk factors. Differences in the number of events were observed between the days immediately prior to hospitalization and 2 weeks before or after hospitalization. While we identified a range of self-care activities and lifestyle risk factors, one feasibility issue was the absence of images of people self-weighing—a common HF self-care practice. This absence could indicate that the study participants did not practice self-weighing or that the forward-facing direction of the camera did not capture the floor scale. Alternatively, as people with HF are typically advised to self-weigh in the morning after toileting, participants may not be wearing the camera during this activity.

Some technical issues warrant discussion. Images were captured every 30 seconds throughout the day, which resulted in approximately 1000 images per participant per day. For this study, we manually reviewed images, which was extremely time-consuming. The quality of the images was mixed, with some images blurred or masked by clothing; thus, we could not code all images due to that lack of clarity. These issues will likely be resolved with technology improvements and participant training protocols. For example, technical solutions and machine learning techniques can review large quantities of data, detect patterns, and filter images into categories for easy recognition and analysis. We are currently applying these techniques to our entire image database.

### Acceptability

Acceptability for using wearable cameras was high for all participants, with most indicating the devices were comfortable to wear and easy to use. While participants self-reported that they thought wearable cameras were acceptable, it was evident from the data that not all participants wore the camera as instructed (during waking hours) every day. While we did not analyze our data to understand this, it could suggest that wearable cameras are only acceptable to participants at certain times of the day or during certain activities.

### Comparison With Previous Research

The high level of acceptability observed in this study was consistent with findings from previous research on older adults older than 65 years with mild cognitive impairment [25]. We also showed that wearable camera images can be used to provide information on sedentary behaviors and physical activities in people with HF, which aligns with previous research on adult university workers [18-20] and children aged 11-13 years [29]. This is the first study, to our knowledge, to use wearable cameras to detect medication practices.

### Strengths and Limitations

The major strength of this study was the novelty of using wearable cameras to observe self-care activities in people with HF. To the best of our knowledge, this is the first study to apply this technology for this purpose. This study resulted in a rich data source, which provided an objective measure of self-care in people with HF. There were limitations to this study. Firstly, a small number of participants was used for analysis; however, as mentioned above, that number of participants yielded 44,000 images for review. Secondly, the time spent in some self-care activities, such as physical activity, is important to understand. In this study, we coded specific events but did not capture the time spent in each activity. Future studies could use automated image processing to analyze the time spent in each activity. Finally, another limitation is that only 1 researcher coded the images, and the intrarater reliability was not assessed; the results need to be considered with this in mind. Future studies should consider 2 or more coders or using automated techniques to code images.

### Implications for Clinical Practice

Wearable cameras offer the potential to help medical and allied health staff better tailor health education for people living with HF. To illustrate, newly diagnosed people with HF might wear a camera for 3-7 days. Automated processing could select or filter images to be rapidly reviewed by a health care professional. These insights into an individual’s self-care practices could then be used to facilitate conversations at outpatient clinics to improve behaviors. Images and conversations might capture misconceptions about self-care advice, such as continuing to add salt to food or purchasing packaged foods that contain added salt. Patient review of images might also increase awareness and motivation for participation in self-care behaviors by providing measures through which progress is tracked. Personalized support driven by the images could be delivered via a user’s smartphone [30]. Moreover, data from wearable cameras could be triangulated with data from cardiac monitors (eg, Holter) or wearable devices (eg, wearable blood pressure monitor or smartwatch) to provide a more holistic and comprehensive view of an individual’s HF condition. Collectively, these data could be used to inform or support clinical decision-making.

### Conclusions

Adults with HF were accepting of using a wearable camera for periods within a 30-day time frame. Wearable cameras were a feasible approach for providing data on selected self-care and lifestyle behaviors for HF. The use of wearable cameras offers the potential to augment clinical practice by tailoring future education for people living with HF; however, issues related to data collection and analysis need to be addressed to improve their utility for this purpose.

## Data Availability

The data sets generated and analyzed during this study are not publicly available as the camera images include identifiable information. However, other data are available from the corresponding author on reasonable request.

## Abbreviations

ADL: activities of daily living
HF: heart failure
